# Evaluation of Health Policy Governance in the Introduction of the New DRG-Based Hospital Payment System from Interviews with Policy Elites in South Korea

**DOI:** 10.3390/ijerph17113757

**Published:** 2020-05-26

**Authors:** Changwoo Shon, Myoungsoon You

**Affiliations:** 1 Department of Urban Society Research, The Seoul Institute, Seoul 06756, Korea; cwshon21c@gmail.com; 2 Department of Public Health Science, School of Public Health, Seoul National University, Seoul 08826, Korea

**Keywords:** good governance, stakeholder participation, payment system reform, DRG-based hospital payment, NVivo 11

## Abstract

The study provides evidence of the governance and its context according to the introduction of the New Diagnosis-Related Groups (DRGs)-based payment system in Korea. In-depth interviews with 14 core policy elites from four health areas were conducted. As governance is a multidimensional concept, interviewees were asked to evaluate different dimensions based on the World Bank’s five elements (Coherent decision-making structures, Consistency and Stability, Stakeholder participation, Supervision and Regulation, and Transparency and Information). Overall, the new payment system was perceived as poorly governed. Since its introduction, it has not offered a new governance perspective because it used a conventional top-down approach, while political windows for cooperation were not wide open. Of the five governance dimensions, the scores were lowest in Stakeholder participation. There was a large perception gap between physicians and government officers here. Participants from academia perceived Consistency and Stability as ineffectively governed. In the meantime, the government has mainly created health policy in Korea. As a result, stakeholder participation, especially the participation of medical personnel, has been insufficient in the process of health policy formulation. The study suggests that the decision-making process in health policy needs to be more participatory and reliable, with governance regarded as a high priority.

## 1. Introduction

Many countries have been reforming their reimbursement system to improve the effectiveness of healthcare services. To successfully reform the payment system, research on the key factors behind successful reform has been carried out [1], finding that governance and stakeholder engagement were the core contributors [2,3]. However, healthcare reform is hard to perfect since each stakeholder has different interests, power levels, and perceptions [4]. In particular, payment system reform is challenging when reforming fee-for-service (FFS) payment to Diagnosis-Related Groups (DRGs) payment because changing payment methods directly influences the revenue of hospitals. For this reason, establishing governance is important, including collaboration with health care providers, consistency in government policies, and public trust in those policies.

While the reimbursement system for hospitals in most countries is based on DRGs, in Korea it is based on fee-for-service. Since the fee-for-service payment mechanism has incentives to increase medical services, it has been pointed out as the main reason for the rise in the cost of national healthcare expenditure, and there has been discussion that the payment system for hospitals should be based on DRGs. However, due to opposition from health care providers, the Ministry of Health and Welfare has implemented a prospective payment system for only seven DRGs since 1997. In 2013, the government introduced the New DRG-based hospital payment system with fee-for-service payment elements to only a few public hospitals.

The subject of this study is to evaluate and understand governance revealed in the process of reforming the hospital’s payment system in South Korea. However, because governance is a conceptual term, it is very difficult to objectively evaluate governance. In particular, there are not many tools for evaluating governance in the course of health policy reform. Our study applied the governance evaluation tool that dealt with the mandatory health insurance system proposed by the World Bank. Since all hospitals in South Korea are subject to the National Health Insurance System, the World Bank‘s tool was considered the most appropriate. The study aimed to provide empirical evidence on “governing health policy” to emphasize the ways in which health policies are formulated, implemented, and evaluated from a political perspective, based on the case of the New DRG-based hospital payment system in Korea.

## 2. Literature Reviews

### 2.1. Governance in Healthcare Fields

The concepts and discussions of governance have been presented in various ways, depending on the scholars and the area and subject of research. There is no universally accepted definition of governance. It remains an elusive theory, defined and conceptualized by various researchers in different fields. Previous studies on governance have provided an overview of how the concept has been used in public administration and public policy, international relations, and comparative politics [5]. In the healthcare sector, the concept of clinical governance was introduced in the United Kingdom in the late 1990s to increase accountability about the quality of patient care [6]. In the 2000s, there was an expansion in health-related financial resources in low- and middle-income countries due to overseas aid such as official development assistance (ODA) [7]. However, donors who demand traceability cut back funding for health during the global financial crisis [8]. In this context, global health governance, one of the core elements in ODA, has been introduced. Studies dealing with global health governance have focused on the evaluation of healthcare needs, corruption of the government and medical providers, and decentralization of the health system; they have also focused on the accountability, efficiency and effectiveness, responsiveness, voice and transparency, leadership, and empowerment of recipient countries [9,10]. In addition, there are three essential elements of global health governance. The first is de-territorialization. As seen in the recent global pandemic of COVID-19, the spread of infectious diseases and population mobility make borders meaningless. The second is to understand the determinants of health in terms of various aspects, such as social environment, physical environment, and natural environment. The third element is that it should formally and informally involve a wider range of actors and stakeholders [11]. Although there are various governance concepts, there is a consensus that the governance function characterizes a set of processes formally or informally applied to distribute responsibility or accountability among actors in a given health system [12].

In terms of collaborative governance, however, a reform based on the top-down approach of the healthcare system or reimbursement system is likely to fail when the stakeholder is excluded from policy formulation [13]. Indeed, a previous study has pointed out that a lack of effective governance between governments, providers, and users is a major cause of health policy reform failure [14]. Although there have been some studies dealing with governance perspective in health policy reform, the majority of governance studies still focus on conceptualization, literature reviews, and suggestions for evaluation tools [15,16,17,18,19,20,21,22,23]. Only a few papers have recently introduced tools to evaluate governance by international organizations [2,24,25,26,27,28,29,30], such as the World Bank measure adopted herein.

According to the five dimensions of the World Bank’s measure, coherent decision-making structures enable those responsible for making decisions to be endowed with the discretion, authority, tools, and resources necessary to fulfill their responsibilities. Stakeholder participation is another core dimension of governance that can influence policy and performance from agenda-setting to the evaluation process. It is rooted in the premise that stakeholder views are integral to meaningful governance and should be incorporated into the decision-making process. Transparency and information ensure that information is available to those who can make decisions. Supervision and regulation are other dimensions of governance that can hold governments accountable for implementing a new payment system. Consistency and stability help avoid uncertainty around rule-making and enforcement, especially during periods of political change. If regulations are consistent, people can make long-term decisions with the assurance that the rules will not change [2]. 

This study focused on the collaborative governance of stakeholders in the decision making and implementation process using the World Bank’s governance concept. Collaborative governance is generally defined such that it is a governing arrangement where public agencies directly engage non-state stakeholders in a collective decision-making process and such that it aims to make or implement public policy [31]. In this context, the governance of this study included the behavior of organizations affected by health policy. For payment system reforms, these factors include its relationship to the government (such as senior officials from the Department of Health or National Health Insurance entities), health care providers (association of medical providers, CEOs or clinicians from the hospitals), and the renowned professors in the health policy field. Ultimately, although citizen participation is also important for collaborative governance [32], in this study, the citizen’s view was not included because there are few channels for citizens to participate in the process of reforming the payment system and since most people are not even interested in the payment system in Korea.

### 2.2. History of the DRG-Based Hospital Payment System in South Korea

The Korean healthcare system has previously used a fee-for-service (FFS) payment scheme for both inpatient and outpatient services since the introduction of social health insurance in 1977. Under the FFS payment system, the insurer focuses on demand-side intervention such as user fees in the form of cost-sharing as a means to reduce healthcare expenditure owing to the excessive utilization of health services. The Korean healthcare system has maintained a high level of copayment. About 41.6% of total expenditure on health comes from private sources in Korea in 2011, making its share of public expenditure far below the average of Organization for Economic Cooperation and Development (OECD) countries. In 2011, 78.4% of private healthcare expenditure was sourced from out-of-pocket expenses [33].

To control the increasing out-of-pocket payment expenditure resulting from the FFS payment system, the government of Korea decided to adopt a DRG payment system like a prospective payment system (PPS) of the US Medicare. Although Korea began to introduce the DRG payment in 1997, it was only applied to a limited number of providers as a pilot initiative due to strong opposition from healthcare providers [34]. Despite continued opposition, however, the DRG payment system was officially adopted for seven DRGs, which included simple procedures and surgeries, in 2002. However, healthcare providers were still given the option to decide whether or not to participate in the DRG payment system. The compulsory adoption of the seven DRGs payment system was only confirmed in 2013, involving all healthcare providers from clinics to general hospitals. At the same time, another DRG payment system, called “the New DRG payment”, similar to the Diagnosis Procedure Combination payment scheme of Japan, was introduced by the National Health Insurance Service (NHIS) to one public hospital in 2009, initially covering 20 DRGs. The number of DRGs and hospitals using this payment method increased every year. The New DRG payment system was applied in 42 public hospitals in Korea, covering 550 DRGs in 2013. As of 2020, 98 hospitals, including private-owned hospitals, are covered by the New DRG payment system on a voluntary participation basis. The New DRG payment system is criticized for having few incentives to reduce medical costs in that it includes considerable elements of fee-for-service payment and allows for the NHIS non-covered items. On the other hand, the opposition from the hospitals exists at the same time because there is a more comprehensive payment system than the previous fee-for-service payment mechanism. The formula for the fee schedule of the New DRG payment system is as follows.

- The New DRG payment fee = Basic point (per diem) * Monetary value per point * Conversion factor (by hospital types) + Unbundled fee (physician services, medication) + Incentives (up to 35% as participation, efficiency, publicness levels)

The NHIS, as a single-payer, is accountable for the payment system including contribution collection and fund management. The NHIS mandatorily contracts to whole medical providers, and every patient can visit any hospital in Korea. That is the reason why most medical providers are sensitive to the reforming of the payment system in Korea. Health Insurance and Review Assessment Service (HIRA) is the regulator with a review and evaluation of medical services. This study will be focused on the accountability of the government, hospitals, and the representative of medical providers through five governance dimensions. These dimensions operate within the context of relationships with the government and medical providers.

## 3. Materials and Methods 

This study was carried out using a mixed-method approach involving both interviews and surveys. For governance evaluation, the assessment was conducted based on the World Bank’s governance evaluation tool by the policy elites, and at the same time, a semi-structured in-depth interview was conducted for the contextual interpretation of the evaluation results. Policy elites can be defined as people who lead the policy-making process and have considerable influence on decision-making. Korea’s health insurance policy is generally led by the government, but in principle, it is formulated through cooperation and discussion from medical providers and experts. In this context, the policy elites included public officers, medical providers, and scholars to explore their perspectives on the New DRG payment system.

Two pilot interviews were conducted before the actual survey to contextualize and modify questionnaires, covering topics about the governance of the New DRG payment system. The Ministry of Health and Welfare (MoHW) recommended two experts as pilot interviewees. One was the healthcare worker from the hospital applying the New DRG payment system. The other one was a senior official from the Health Insurance Review and Assessment Service (HIRA).

After that, a snowball technique was used to select the interviewee. We asked each interviewee to recommend five powerful policy elites in health policy as potential interviewees. This technique allowed us to select interviewees that were referred to by respondents, including the pilot interviewees. A total of 14 core policy elites were selected and interviewed from January to July in 2013. In principle, we conducted the governance evaluation through mini-survey and in-depth interview once. However, if an additional contextual interpretation was needed, it was supplemented through a telephone interview after that. Government interviewees included high-ranking officials from the MoHW, HIRA, and NHIS, and those of physician groups included representatives from the Korean Hospital Association (KHA) and Korean Medical Association (KMA), which represent medical provider groups. The interviewees from academia included scholars from different colleges of health or medical sciences. In addition, a category for hospitals that use the New DRG payment system was added, including the CEOs and chiefs of such hospitals.

The general characteristics of the interviewees are shown in Table 1. The authors conducted all interviews and surveys directly, and all interviewees agreed that the interview script could be used for research by signing the document and recording a voice. Interviews lasted around 90–120 minutes and focused on following the World Bank’s five governance dimensions: Coherent decision-making structures, Consistency and Stability, Stakeholder participation, Supervision and Regulation, and Transparency and Information (Appendix A).

Based on the World Bank’s governance tool, we contextualized and specified the features in the original frame to make them more understandable to South Korea’s payment system. In order to enhance the validity of the evaluation tool, the research was carried out after receiving consent and advice from Savedoff through E-mail—the developer of the tool—on the utilization of the assessment tool and the modification of the assessment tool. The evaluation tool consisted of 12 questions. For five dimensions, we conducted a seven-point Likert scale to show governance structures by visualization and regarded more than four points as a positive evaluation. First, we identified the *Coherent decision-making structures* dimension, which included three questions on whether (i) all the institutions in charge of the supervision of payments can fulfill their responsibilities, (ii) they have routines for risk assessments and management strategies in place, and (iii) the cost of regulating and administering them is reasonable and appropriate. Second, we evaluated the *Stakeholder participation* dimension with a single question on whether each stakeholder has effective representation in the governing bodies in terms of introducing, implementing, and assessing the new payment system. Third, we explored the *Transparency and Information* dimension by using four questions on whether (i) the objectives of the new payment are formally and clearly defined; (ii) the main components of the new payment system rely upon an explicit and appropriately designed institutional and legal framework; (iii) clear information, disclosure, and transparency rules are in place; and (iv) the new system has minimum requirements to protect patients such as the protection of privacy or personal information. Fourth, the main feature of the *Consistency and Stability* dimension is the quality of the system. To identify the stability of the system, we asked whether the purpose and motivation of the new payment system remained substantially the same during past political change. Lastly, we evaluated the *Supervision and Regulation* dimension by using three questions on whether (i) the rules on compliance, enforcement, and sanctions for the new payment supervision are clearly defined; (ii) the financial management rules for hospitals are clearly defined and enforced; and (iii) the new payment system has structures for ongoing supervision and monitoring in place. Gaps in health policy governance were explored in accordance with the identified governance indicators.

At the end of the interviews, we added items about the governance of Korea’s new payment system to the assessment of the overall evaluation that were not in the original frame of the World Bank. The assessment of overall evaluation was carried out using a survey, which included the following two questions.

- Please score the overall evaluation in terms of the introduction, implementation, and assessment of the new payment system from good governance perspectives (out of 10 points).
- If the new payment system is unchanged (none of its components change), do you think it could remain a successful healthcare policy? (out of 10 points).

**Figure 1:**
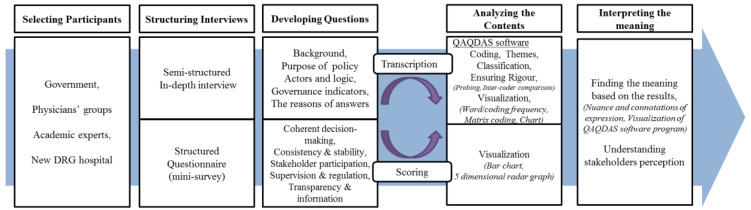
Research process steps: An evaluation of the perception of governance.

For an in-depth interview, the transcripts were coded and thematically analyzed by using NVivo 11 software, and the results were displayed in a five-dimensional graph to conceptualize the relative significance of each dimension. We used a systematic approach to the analysis that broadly involved familiarization, the identification of the framework, indexing, charting, and interpretation [35]. We provided independent coding and analysis to cross-check themes and interpret the whole dataset. The study also received approval from the Seoul National University Institutional Review Board (IRB No. 51-2012-11-30). The research process for evaluating governance in health policy is described in Figure 1. 

## 4. Results

### 4.1. Participants

The majority of participants were male (N = 9), and the age group most represented was between 45 and 55 years (50%). The minimum age of participants was 39 years, and the maximum age was 60 years, with a mean age of 51.2 years. The majority of participants with medical licenses were medical doctors (N = 6), and the rest were nurses or medical record technicians. Most respondents had worked for more than 10 years and had medical licenses. The general characteristics of the participants are described in Table 1.

### 4.2. Analysis of Coding

This study applied a bottom-up integration method that narrowed the subcategories and main categories by using computer-assisted qualitative data analysis. From the initial codes, we were able to merge and develop clustered codes for analysis. After cross-checking, we drew analyzable codes composed of five major categories and 31 subcategories (Appendix B). The major categories are Coherent decision-making structures, Consistency and Stability, Stakeholder participation, Supervision and Regulation, and Transparency and Information. In the area of coherent decision-making structures, eight subcategories were derived, including HIRA internal conflict and the conflict with the MoHW. In the Consistency and Stability area, three subcategories, including the absence of a law for the New DRG payment system, were drawn, and in the stakeholder participation area, seven subcategories, including the lack of previous consensus-building experience, were derived. In the Supervision and Regulation area, four subcategories, including the lack of monitoring indicators and capacity, were found, and in the Transparency and Information area, nine subcategories, such as distrust of each research result and the low accessibility of information access, were derived. To develop coding reliability, we conducted a coder comparison with a Ph.D. candidate in the healthcare field using Kappa values. Our measure of inter-observer variation has been used elsewhere [36]. The Kappa value for this study was found to be about 0.68, which is a substantial reliability value. Generally, a kappa coefficient of > 0.4 means moderate, > 0.6 means substantial, and 0.8 means almost perfect [37].

The frequencies of the categories brought up by participants are described in Appendix C. Government interviewees mainly referred to Coherent decision-making structures and Stakeholder participation, while medical providers referred to Transparency and Information the most. Like medical providers, hospitals mentioned Transparency and Information and Stakeholder participation among the five dimensions, whereas scholars often mentioned Coherent decision-making structures and Consistency and Stability. In brief, government participants were interested in exploring the capacity of hospitals and stakeholder participation, while medical providers including hospitals that use the New DRG-based payment system emphasized participation and transparency. Furthermore, academia pointed out the balance of power and capacity between governments and DRG hospitals.

### 4.3. Evaluation of the New DRG-Based Payment System from a Good Governance Perspective

The good governance of the New DRG payment system was generally defined as a fair rule of the game in the implementation process. This included (i) the process by which those with authority are selected, monitored, and replaced; (ii) the capacity of the government to effectively manage its resources and implement sound policies; and (iii) the respect of citizens and the state for the institutions that govern economic and social interactions among them [38]. From this perspective, we asked participants to score (on a 10-point scale) the rule of exercising power and managing conflicts under the New DRG payment system.

**Figure 2:**
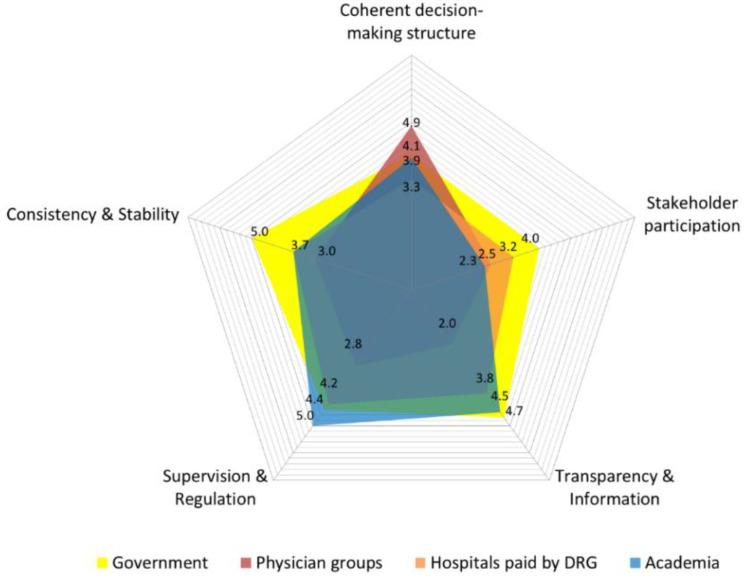
Governance performance: assessment by stakeholders.

Based on the survey, the overall evaluation of participants is described in Figure 2. The government scored higher (6.3) than any other stakeholders (academia 4.3, hospitals 3.8, physicians 2.5). More importantly, the score of perceived governance by government representatives was more than twice as large as that from the hospitals that use the New DRG system. There is thus a huge gap in perception between government bodies and physician groups according to the results.

We also asked stakeholders to assess the prospects of the New DRG payment system by using a 10-point scale. Most stakeholders set a low estimation of the successful implementation of the New DRG payment system. The government again scored higher (5.3) than the other stakeholders (academia 4.7, physicians 3.5, hospitals 3.0). The remarkable result of this survey was that the perception of hospitals that use the New DRG system was worse than that of physician groups. Generally, in Korea, when a new regulatory policy is implemented, medical organizations such as KMA are more strongly opposed than individual doctors. However, in the case of the New DRG payment, it was somewhat surprising that the individual doctors who experienced the system had more objections than medical organizations.

### 4.4. Evaluation of the Five Governance Dimensions

Participants’ evaluations of each governance dimension were inconsistent. First, in terms of the *Coherent decision-making structures* dimension, the perception gap between stakeholders was at its narrowest. Physician groups (4.9) and government representatives (4.1) perceived the decision-making structures in the governance of the New DRG-based payment system as relatively coherent, whereas academia (3.9) and hospitals (3.3) did not. Second, *Stakeholder participation* was the weakest dimension in Korea’s new payment system (government 4.0, hospitals 3.2, academia 2.3, physician groups 2.5). Although the government evaluated this dimension positively compared with the other stakeholders, this dimension was evaluated the lowest among all governance structures. However, the perception gap among participants was not that large because most also perceived this dimension to be the worst. Third, regarding *Transparency and Information*, government (4.7) and academia (4.5) participants perceived this dimension to be relatively well-made, while physician groups (2.0) and hospitals (3.8) evaluated it negatively. In particular, a huge perception gap between the government and physician groups was revealed in this dimension. Fourth, relative to the other dimensions, *Supervision and Regulation* performance was quite good except for physician groups (governments 4.4, hospitals 4.2, academia 5.0). Only physician groups evaluated this dimension in a negative way (2.8). Finally, other aspects of *Consistency and Stability* showed similar weaknesses to stakeholder participation (physician groups 3.0, hospitals 3.7, academia 3.7). On the contrary, the government evaluated this dimension (5.0) as the best structure among the five dimensions (Figure 2).

### 4.5. Findings from the In-Depth Interviews

#### 4.5.1. Coherent Decision-Making Structures

There was a perception gap between hospitals that use the New DRG system and physician groups. Hospitals and academia perceived that the imbalance between the MoHW and HIRA harmed coherent decision-making structures. In other words, only the MoHW has the power to decide on a new payment system even though HIRA planned, implemented, and evaluated it at a working level.

“I feel bad (pity) for HIRA. HIRA reviewed and evaluated the hospital system, and they do all the practical work of health policy from the MoHW. HIRA gets commissioned most of the health policy works from the MoHW. (But they don’t have any authority to modify policies)”.

(No. 3)

“When we offer opinions to HIRA, shouldn’t they apply health policy based on what we tell them? When HIRA comes to our hospital, they pay attention and listen to what we say or suggest. HIRA also says that our opinion is something that they could use, but that’s all (they don’t move forward towards changing the health policy)”.

(No. 10)

Hospitals that use the New DRG system pointed out the lack of skilled human resources and equipment; they asked the government to increase the budget for public hospitals using the new payment system. One academic interviewee also emphasized the increasing fee schedule for setting up hospital administration under the DRG payment system.

“The administrative burden of the New DRG payment system goes to the hospitals, and the government should raise the total medical expenses (fees) to keep up with the new system. But the problem is they don’t”.

(No. 14)

#### 4.5.2. Stakeholder Participation

The dimension of stakeholder participation was the weakest; most stakeholders except the government mentioned a variety of reasons why this is a pressing problem. In academia, the indifference of people towards health policy was one of the reasons, as well as the government’s policy blindly following the political tendency of the KMA executives.

“Citizens are usually not aware of the payment system, and they don’t care. This is the main issue why the participation of the stakeholders is not vigorous”.

(No. 13)

“There’s a new executive department with an extremely progressive attitude in the KMA, so they usually go against the government’s idea without even looking at the new system”.

(No. 12)

On the contrary, hospitals that use the New DRG system perceived that the government rarely considers such hospitals’ views in decision making. Although there is consensus about the structure of the policy reform, they have not experienced agreement before. Moreover, hospitals need feedback even though their opinions are not reflected in the modified policy.

“The MoHW formally sends a survey to the doctors, and accept the opinion that they won’t apply. They even notify that the New DRG is good enough since they accepted the doctors’ opinions”.

(No. 9)

We had a lot of meetings. We told the MoHW several times about the new system’s problems from the clinicians’ view (emphasized) but they still don’t listen to us. It was nothing more than empty talk”.

(No. 10)

Physician groups focused on the importance of trust in medical doctors. They noted that clinical medical doctors should participate in consensus building as a partner.

“Doctors never use tricks to make more profits or do bubbling (to exaggerate to make money). Why don’t they use actual clinicians who are ethically qualified? Doctors who are college professors or who are in the health professional associations are… (they don’t represent the actual practitioners)”.

(No. 5)

#### 4.5.3. Transparency and Information 

Physician groups pointed out the trust gap between medical doctors and the government. They perceived that the government used to push policy unilaterally without an agreement. Besides, they thought the government had hidden messages behind the objectives of the New DRG payment system, and they did not trust the research results from either side.

“The people from NHIC or those who are involved in health policy don’t usually trust the doctors. Maybe they have experienced few doctors’ immortal behaviors or such things to cause social problems…”.

(No. 10)

“If the payer provides more money than under the FFS, then the government and citizens won’t agree with it, and if doctors are provided less, then they won’t accept it. I am sure the MoHW will give enough budgets for the first few times to attract us (doctors), and then decrease as the system settles down”.

(No. 4)

#### 4.5.4. Supervision and Regulation

This dimension was the strongest among the five dimensions, with most stakeholders except medical providers perceiving few problems with the government’s regulation. Physician groups emphasized that the quality indicators suggested by the government are inappropriate. They also insisted that the government should consider different contexts of DRG payment due to health reform rights because they perceived that the government has slackened its effort to understand other countries’ DRG payment systems.

“The health statistics provided by the government are just a joke to trick the doctors and citizens. Of course, the quality of care will decrease. The form of what they evaluate is ridiculous”.

(No. 4)

“In America, they separate the doctor’s fee and hospital management fee, and the Korean MoHW just tries to copy the U.S. DRG system. How pathetic this situation is when the people from the MoHW don’t even know the Korean system and just try to use the U.S. system without any consideration”.

(No. 5)

#### 4.5.5. Consistency and Stability

Consistency and stability was the second weakest dimension. Most stakeholders except the government perceived that the government has serious problems owing to the discontinuity of policy. There is no coherence to government policies because of job rotation and public officials’ preference towards public organizations. For this reason, the government and academic participants pointed out the need for a National Assembly Act related to payment systems. They also underlined an independent organization, instead of HIRA, responsible for payment is required to establish governance consistency.

“The people who work for the MoHW in a higher position usually don’t work for more than two years. A lot of public officials usually follow only the end of the period system when the new chief is selected…”.

(No. 5)

“The New DRG system actually can change at any time depending on the chief or who is in a high position in the MoHW”.

(No. 12)

As a result of the interviews, the governance evaluation of the government and the medical providers was significantly different in five dimensions of governance. In particular, it is natural that doctors have a different voice from the government on the introduction of the DRG payment system due to medical doctors having a great deal of autonomy as professionals. However, the problem is that these differences in the assessment of the components of the governance structure do not result from differences in positions such as clinical experts and administrators, but from lack of trust. Governance perception of the medical provider group was even worse than clinical doctors in hospitals, and they doubted that the government’s introduction of DRG is the first step towards the global budgeting system. The main findings from these in-depth interviews are summarized in Table 2.

## 5. Discussion

The Korean healthcare system, especially its payment system, has been reformed with the introduction of a new DRG-based payment scheme. In this study, we conducted in-depth interviews and structured surveys with policy elites to show the importance of governance in health policymaking by analyzing the perception gaps of stakeholders in Korea. We used the snowball sampling method because policy elites related to the payment system have not previously been identified and are more difficult to locate or contact [39]. 

In Korea, typical health policy stakeholders include government bodies, physician groups, and academia. Indeed, the government and the payer play a key role in a health insurance policy by regulating and managing social health insurance, setting fee levels to be paid to the healthcare provider, and setting benefit coverage [40]. Physician groups are also considered to be a core stakeholder that has clinical expertise in the health system, as in other countries. Scholars influence health policy through the results of their research; however, civil organizations have less influence on the health system in Korea. 

The government has played a leading role in reforming the payment system in Korea [41]. In particular, the government tried to persuade medical providers by developing a more sophisticated reimbursement model using claim data analysis. However, the resistance of medical providers to the introduction of the New DRG payment system has still existed. To look at the problem differently, we emphasized the importance of the governance concept that has not been noticed during health reform in Korea. To measure the governance of the New DRG payment system, this study adopted in-depth interviews and a survey to assess the perception gaps between key stakeholders related to governance issues using the approach by the World Bank. However, the governance concept between Western and Eastern countries may be different due to different contexts. For example, civil society, one of the core dimensions of governance, is wholly new in Asian countries, including Korea [42]. Nevertheless, with the lack of international consensus on governance evaluation, the World Bank’s approach has enabled empirical research on policy governance.

This study is the first empirical study of health system governance in Korea. This empirical study is meaningful because it revealed distinct pictures of health policy governance outside of the Western countries. For this, we asked policy elites to evaluate the New DRG payment system from a governance perspective to show the perception gaps in five dimensions. 

As the results of the evaluation, among the components of governance, a dimension of Participation and Consistency and Stability was the most vulnerable elements. All other stakeholders except the government evaluated the policy consistency as low. Considering that the hospital’s score was the lowest, it seems that the opinions of the clinicians of hospitals were not reflected during the payment system reform. Furthermore, in most of the components, we found significant differences in perceptions between the government and medical providers, including the hospitals. In particular, policy elites pointed out that the issue of stakeholder participation was the biggest problem among the five governance components of the New DRG payment system. In terms of collaborative governance, the lack of participation by experts in making policy and implementation process is a flaw in Korea. In other words, the huge challenge in payment reform in Korea appears to be not only inexact modeling and affordability but also stakeholder participation and political consistency. It is important that the government shares the policy goal and its values with other stakeholders and that efforts to involve them in the decision-making process are essential. These points are supported by previous studies that have found that consensus and political feasibility are key factors to reforms, rather than the sustainability of financing [15]. We confirmed that the key success factor for payment reform in the Korean context is strengthening the governance structure and building consensus. 

However, the findings of this study should be interpreted with the following limitations. First, selection bias exists because of the small sample size [43], even though the interviewees were selected from a large number of recommendations by each participant to minimize such bias. Considering the limited generalizability, this study still provides a better understanding and more complete characterization of the health-related views of policy elites in Korea. Second, the validity of survey responses could be low because of social desirability bias, which is when interview participants tend to avoid negative opinions or embarrassing comments about themselves or their organizations [44]. Third, the rigorousness of the governance evaluation tool, the World Bank’ suggested, can be relatively low, although the survey questionnaire was modified by the experts’ consultations and preliminary interviews.

Despite these limitations, this study suggests that health policy elites should focus on not only the sufficiency of financial support but also a good governance structure in the health system. The road to collaborative governance in health policy is long and uneven. Assessing health policy by using governance indicators is only the first step towards improving the overall system [18]. Therefore, efforts to improve governance should be deliberate from making to implementing health policy.

## 6. Conclusions

In this study, most participants, except the government, showed a low value in the overall evaluation of the new payment system from the identified good governance perspective. Unfortunately, the perception of hospitals that use the New DRG payment is even worse than that of physician groups regarding the probability of the successful establishment of the new payment system. The government’s perception of all five dimensions of governance was positive. Physician groups perceived low governance in most dimensions except for Coherent decision-making structures. In particular, as they evaluated lower in four dimensions, we found a huge perception gap between the government and physician groups. The tendency of hospitals that use the New DRG system was similar to that of physician groups; however, they evaluated the Stakeholder participation dimension lower than physician groups. Academia also evaluated most dimensions low, except for the Transparency and Information dimension.

In the meantime, there have been many quantitative studies using hospital data to reform the DRG payment system in Korea. But it still has not been successfully settled. In this context, this study presented another perspective on payment system reform by re-evaluating the New DRG payment system through the emerging concept of governance. Particularly, in Korea, efforts should be made to recognize doctors as partners of the government and to participate in the policymaking process. This study also provided insights to understand why health reforms must consider collaborative governance among stakeholders. This study has also contributed to bridging the gap between the theory and reality by applying governance evaluation tools. Policy elites should understand the reasons behind the gaps in each governance dimension to ensure successful payment reform.

## Figures and Tables

**Table 1:** General characteristics of participants.

ID No.	Institution	Position	Gender	Medical License	Years Spent in Current Position ^1^
1	Government	Head of dept.	Female	Nurse	Long
2		Deputy director	Male	Doctor	Recent
3		Director	Male	No	Long
4	Physicians	Executive of the KHA ^2^/ CEO of a public agency	Male	Doctor	Recent
5		Executive of the KMA ^3^/ CEO of a general hospital	Male	Doctor	Long
6	Hospitals using the New DRG Payment system	CEO	Male	Doctor	Long
7		Chief of dept.	Female	Medical record technician	Long
8		Chief of dept.	Female	Nurse	Long
9		Chief of dept.	Female	Nurse	Long
10		Chief of dept.	Male	Doctor	Long
11		Vice-president	Male	Doctor	Long
12	Academia	Scholar (School of Medicine)	Male	Doctor	Long
13		Scholar (Graduate School of Technology Management)	Female	No	Recent
14		Scholar (School of Public Health)	Male	No	Long

^1^ Under 10 years: Recent, over 10 years: Long; ^2^ Korean Hospital Association; ^3^ Korean Medical Association.

**Table 2:** Summary of the main findings by each governance dimension.

Governance Dimension	Main Findings
Coherent decision-making structures	There was a gap between decision-making power and capacity in government bodies. There was a lack of skilled human resources and equipment in hospitals that use the New DRG-based system.
Stakeholder participation	There was a seriously poor performance compared with what we expected. People were indifferent to health policy. Doctors who are actually treating patients were excluded from the implementation process.
Transparency and Information	There was distrust between medical doctors and the government. Medical doctors believed that the government had hidden messages behind the objectives of the DRG payment system.
Supervision and Regulation	Medical doctors needed to revise inappropriate quality indicators. Medical doctors wanted the government to understand the different contexts of other countries’ cases.
Consistency and Stability	The instability of the payment system was revealed due to job rotation in public sectors. Health policies were often decided according to high-ranking officials’ preferences.

**Figure A1:**
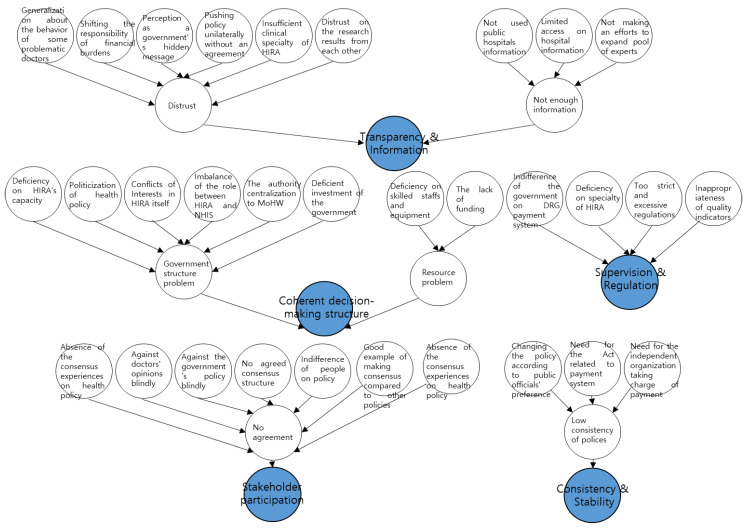
Five major categories and 31 subcategories.

**Figure A2:**
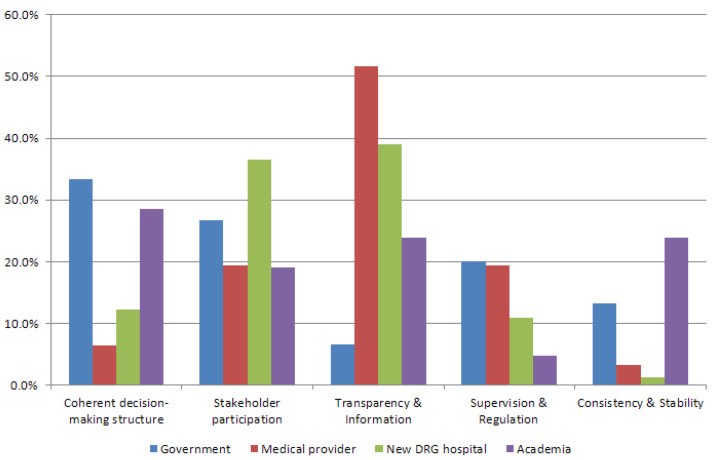
Coding frequency (%) by stakeholders.

## Appendix A. Interview Questionnaires: Governance of the New DRG-Based Hospital Payment

I. Main Survey (World Bank Governance Evaluation Tool)

**● Coherent decision-making structure**

1. Does responsibility for the New DRG-based payment system objectives correspond with decision making power and capacity in each institution involved in the management of the system?

**Strongly Disagree**	**Disagree**	**Somewhat Disagree**	**Neutral**	**Somewhat Agree**	**Agree**	**Strongly Agree**	**n/a**
1	2	3	4	5	6	7	

2. Do all New DRG-based payment system entities have routine risk assessment and management strategies in place?

**Strongly Disagree**	**Disagree**	**Somewhat Disagree**	**Neutral**	**Somewhat Agree**	**Agree**	**Strongly Agree**	**n/a**
1	2	3	4	5	6	7	

3. Is the cost of regulating and administering the New DRG-based payment system reasonable and appropriate?

**Strongly Disagree**	**Disagree**	**Somewhat Disagree**	**Neutral**	**Somewhat Agree**	**Agree**	**Strongly Agree**	**n/a**
1	2	3	4	5	6	7	

**● Stakeholder participation**

4. Do stakeholders have effective representation in the governing bodies of the New DRG-based payment system entities?

**Strongly Disagree**	**Disagree**	**Somewhat Disagree**	**Neutral**	**Somewhat Agree**	**Agree**	**Strongly Agree**	**n/a**
1	2	3	4	5	6	7	

**● Transparency and Information**

5. Are the objectives of the New DRG-based payment system formally and clearly defined?

**Strongly Disagree**	**Disagree**	**Somewhat Disagree**	**Neutral**	**Somewhat Agree**	**Agree**	**Strongly Agree**	**n/a**
1	2	3	4	5	6	7	

6. Does the New DRG-based system rely upon an explicit and appropriately designed institutional and legal framework?

**Strongly Disagree**	**Disagree**	**Somewhat Disagree**	**Neutral**	**Somewhat Agree**	**Agree**	**Strongly Agree**	**n/a**
1	2	3	4	5	6	7	

7. Are clear information, disclosure, and transparency rules related to the New DRG-based payment system in place?

**Strongly Disagree**	**Disagree**	**Somewhat Disagree**	**Neutral**	**Somewhat Agree**	**Agree**	**Strongly Agree**	**n/a**
1	2	3	4	5	6	7	

8. Do the New DRG-based payment system entities have minimum requirements (including consumer information, and independent mechanisms for the resolution of complaints, appeals, grievances, and disputes) regarding protecting patients?

**Strongly Disagree**	**Disagree**	**Somewhat Disagree**	**Neutral**	**Somewhat Agree**	**Agree**	**Strongly Agree**	**n/a**
1	2	3	4	5	6	7	

**● Supervision and Regulation**

9. Are the rules on compliance, enforcement, and sanctions for supervision clearly defined?

**Strongly Disagree**	**Disagree**	**Somewhat Disagree**	**Neutral**	**Somewhat Agree**	**Agree**	**Strongly agrEe**	**n/a**
1	2	3	4	5	6	7	

10. Are financial management rules for the New DRG-based payment system clearly defined and enforced in legal text or regulations?

**Strongly Disagree**	**Disagree**	**Somewhat Disagree**	**Neutral**	**Somewhat Agree**	**Agree**	**Strongly Agree**	**n/a**
1	2	3	4	5	6	7	

11. Does the New DRG-based payment system has structures for ongoing supervision and monitoring in place?

**Strongly Disagree**	**Disagree**	**Somewhat Disagree**	**Neutral**	**Somewhat Agree**	**Agree**	**Strongly Agree**	**n/a**
1	2	3	4	5	6	7	

**● Consistency and Stability**

12. Are the main qualities of the New DRG-based payment system stable? (i.e. independent of political elections or economic crises).

**Strongly Disagree**	**Disagree**	**Somewhat Disagree**	**Neutral**	**Somewhat Agree**	**Agree**	**Strongly Agree**	**n/a**
1	2	3	4	5	6	7	

**● Overall evaluation**

13. Please score the overall evaluation in terms of the introduction, implementation, and assessment of the new payment system from good governance perspectives.

**← Lowest Score Highest Score→**										**n/a**
1	2	3	4	5	6	7	8	9	10	

14. If the new payment system is unchanged (none of its components change), do you think it could remain a successful healthcare policy?

**← Lowest Score Highest Score→**										**n/a**
1	2	3	4	5	6	7	8	9	10	

**- Thank you**
